# Evaluating Sequence Foundation Models for Insect Olfactory Gene Discovery: Failure Modes, Evidence Standards, and a Benchmark Agenda

**DOI:** 10.3390/insects17090928

**Published:** 2026-09-04

**Authors:** Huiqin Li, Dian Zhou, Wei Pu, Guoxing Wu, Chun Xiao, Xi Gao, Junfu Yu

**Affiliations:** 1 State Key Laboratory for Conservation and Utilization of Bio-Resources in Yunnan, Yunnan Agricultural University, Kunming 650201, China; lihuiqin0809@163.com (H.L.); wugx1@163.com (G.W.); x.chun@163.com (C.X.); 2 College of Plant Protection, Yunnan Agricultural University, Kunming 650201, China; 3 College of Sanqi Medicine and Pharmacy, Wenshan University, Wenshan 663000, China; zhoudian_hn@163.com; 4 School of Continuing Education, Honghe University, Mengzi 661199, China; weipu_uoh@163.com; 5 College of Big Data, Yunnan Agricultural University, Kunming 650201, China

**Keywords:** insect olfaction, insect chemosensation, olfactory gene discovery, sequence foundation model, protein language model, DNA foundation model, gene annotation, evidence standard, benchmark, cross-species transfer

## Abstract

Genome-scale searches for insect olfactory genes are vulnerable to rapid receptor divergence, tandem duplication, fragmented gene models, circular labels, and evolutionary leakage between training and test data. Sequence foundation models trained on DNA or proteins may extend homology-based annotation, but available evidence does not support their use as autonomous annotators or direct predictors of sensory function. This critical review organizes the problem around annotation failures rather than model catalogues. We distinguish six inference levels, from candidate locus detection to organism-level function, and define task-specific high-confidence reference criteria. We also propose a benchmark using biological hard negatives, sequence-cluster and taxonomic holdouts, conventional baselines, calibration, abstention, and workload-aware retrieval metrics. Evidence integration is defined operationally as a pipeline that retains model scores alongside similarity, domains, topology, phylogeny, expression, and assay evidence, without promoting a weak signal to a stronger biological claim. A published ESM-2-supported analysis of remote insect chemoreceptor homology illustrates both the potential and the current evidence boundary. A convincing benefit will require an incremental gain over transparent baselines under frozen biologically realistic splits, with reliable uncertainty and a clear route from ambiguous predictions to review or experiment.

## Simple Summary

Insects rely on smell to locate food, hosts, mates, and egg-laying sites, making olfactory genes useful targets for ecological and pest management research. These genes are difficult to identify because many evolve rapidly, occur in duplicated clusters, or are represented by incomplete gene models. Sequence foundation models trained on DNA or proteins may help recover candidates missed by conventional searches, but a high score does not establish family identity or sensory function. This review links common biological and computational failure modes to the evidence required for each claim. It also proposes a benchmark that compares sequence models with similarity searches, profile HMMs, phylogeny, and simple machine learning baselines on hard negatives and evolution-aware test sets. The most credible near-term role of SFMs is to rank uncertain candidates and to route difficult cases to expert review.

## 1. Introduction

Olfaction allows insects to respond to intermittent chemical cues from hosts, food, mates, oviposition sites, competitors, and predators. These responses influence crop damage, vector–host contact, biological invasion, pollination, and community interactions. The underlying molecular system includes odorant receptors (ORs), ionotropic receptors (IRs), gustatory receptors (GRs) with olfactory roles, odorant-binding proteins (OBPs), chemosensory proteins (CSPs), sensory neuron membrane proteins (SNMPs), and several enzyme families implicated in odorant degradation [1,2,3,4,5,6,7]. Receptors and their ligands are also relevant to surveillance lures, mating disruption, attract-and-kill strategies, and repellents [2,3,8,9].

The phrase “finding olfactory genes” nevertheless combines distinct inference problems. A plausible coding region may still have the wrong family assignment, and a reliable family assignment does not establish orthology, sensory tissue expression, ligand response, or a causal role in behavior. Each claim requires a different reference standard. For example, an OR-like sequence on a fragmented scaffold is a candidate locus, not a validated receptor for a host volatile. Antennal enrichment can prioritize a soluble protein or enzyme, but it cannot establish odor transport or degradation. Annotation becomes misleading when evidence supporting one level is used to justify a stronger claim.

Conventional comparative genomics remains the starting point. BLAST or DIAMOND searches, profile hidden Markov models (HMMs), curated domains, topology and signal peptide prediction, multiple-sequence alignment, phylogenetic placement, transcript support, and manual locus inspection provide evidence that a curator can trace [10,11,12,13,14,15,16,17,18,19,20]. These methods work especially well for conserved co-receptors and families with broad reference coverage. Their sensitivity is less predictable for lineage-specific tuning receptors, recent duplicates, partial genes, and remote homologs, which are often the sequences of greatest ecological interest. The recently described connection between insect chemoreceptors and a wider cryptic protein superfamily illustrates both the biological value and the difficulty of detecting deep homology [21].

Sequence foundation model (SFM) is used here as the umbrella term for pretrained models that learn representations from biological sequences. Protein language models (PLMs), such as ESM and ProtT5, operate on amino acid sequences and are naturally aligned with protein-family retrieval and structural constraint [22,23,24]. DNA foundation models, including DNABERT, DNABERT-2, nucleotide transformer, HyenaDNA, GENA-LM, Evo, and Evo 2, operate on nucleotide sequence and can represent coding grammar, splice signals, and genomic context [25,26,27,28,29,30,31,32,33]. Thus, PLMs and DNA foundation models are two SFM branches rather than interchangeable names. Their pretraining objectives usually reconstruct masked or next sequence elements; task-specific classifiers or retrieval systems then learn labels such as family membership, genomic element type, or assay outcome.

Broad pretraining may improve weak-candidate retrieval or transfer to sparsely annotated species, but it also introduces computational cost, opaque feature use, uncertain pretraining overlap, and sensitivity to the difference between training and target data. We call this difference a domain shift: for example, a model trained mainly on vertebrate or microbial sequences is applied to a divergent insect order with different composition, gene-family dynamics, and annotation conventions. General genomic benchmarks do not show universal gains, and tuned conventional or supervised models have matched or exceeded frozen representations in several tasks [34,35,36,37,38]. The relevant question is therefore not whether SFMs are technically capable but whether they improve a defined decision beyond strong transparent baselines.

This article is a critical review and benchmark agenda, not a systematic review or a completed model comparison. We updated the literature through 20 August 2026 using targeted PubMed and publisher database searches, primary model and benchmark papers, and backward and forward citation checking. A title/abstract search combining SFM or PLM terms with insect and olfactory terms found no peer-reviewed head-to-head comparison of a PLM or DNA foundation model with BLASTp and HMMER for insect olfactory-family annotation. Full-text review did identify an ESM-2-supported analysis of remote insect chemoreceptor homology [21], discussed below. Because the search was not preregistered, independently screened, or designed as a PRISMA review, we make no claim of exhaustive coverage. We instead organize the field around three questions: where olfactory gene-mining pipelines fail, what evidence each biological claim requires, and how a benchmark could test whether an SFM adds practical value.

## 2. Biological and Annotation Challenges Define the Evaluation Task

### 2.1. Gene Families Create Different Decision Boundaries

Insect ORs are a large and rapidly evolving family of seven-transmembrane ion-channel subunits. Most odor-specific ORs assemble with the conserved co-receptor Orco, although basal lineages and divergent receptors reveal a more complex evolutionary history [21,39,40,41,42,43,44,45,46,47,48]. Orco is usually straightforward to retrieve because it is conserved. Tuning ORs are harder because duplication and loss create lineage-specific repertoires with few simple one-to-one relationships across distant orders. A benchmark dominated by conserved representatives can therefore report high accuracy while missing the copies most likely to mediate ecological specialization.

IRs create a different boundary. Chemosensory IRs evolved from ionotropic glutamate receptors (iGluRs), retain family-level structural relationships, and include both conserved co-receptors and divergent tuning subunits [1,4,5]. A model may learn broad iGluR-like properties without resolving whether a sequence is a synaptic iGluR, a canonical antennal IR, or a lineage-specific chemosensory branch. The difficult negatives are consequently close relatives that share domains and architecture, not randomly sampled proteins. Phylogenetic placement forms part of the reference label rather than a decorative downstream analysis.

Soluble and accessory proteins add further ambiguity. OBPs and CSPs are often retrieved using signal peptides, cysteine patterns, domains, expression, and similarity to curated references [7,18,49]. These features are informative but not decisive in isolation. Partial sequences may lose a diagnostic cysteine, and atypical members may violate simple length filters. SNMPs belong to the wider CD36-related family, so CD36-family membership does not establish an olfactory role [50,51,52]. “Odorant-degrading enzyme” (ODE) is a functional umbrella that includes esterases, cytochrome P450s, aldehyde oxidases, glutathione transferases, and other enzyme groups [53,54]. An ODE benchmark must therefore keep the enzyme-family label separate from evidence of odorant degradation.

These boundaries define different tasks: OR versus GR, chemosensory IR versus conserved iGluR, OBP versus CSP, olfactory SNMP versus another CD36 protein, and an odorant-associated enzyme versus a metabolic paralog. Informative negatives and supporting evidence change with each task. Combining all families in one multiclass score can make a model look successful because it recognizes easy classes while failing at the biologically important boundaries.

### 2.2. Evolutionary Relatedness Invalidates Convenient Random Splits

Chemosensory repertoires evolve through duplication, divergence, pseudogenization, gene loss, and lineage-specific expansion [6,48,55,56,57,58,59,60]. Tandem arrays are common, and recent paralogs may be far more similar to one another than to sequences in another species. A random split assigns individual records to training, validation, or test sets without respecting this relatedness. If members of one recent cluster occur in both training and test sets, the evaluation measures interpolation among near duplicates rather than discovery in a new genome.

Random splitting of evolutionarily related sequences creates taxonomic leakage and shortcut learning. Taxonomic leakage occurs when closely related taxa, or proteins copied through the same annotation pipeline, appear on both sides of the split. Shortcut learning occurs when a classifier exploits correlated features, such as length, hydrophobicity, amino acid composition, codon usage, or annotation conventions, instead of the intended family boundary. Some of these features are biologically meaningful, but none alone establishes the target claim. An OBP classifier driven mainly by length can fail on partial or atypical proteins, while a DNA model may learn taxon-specific codon composition rather than gene-family identity.

Evolutionary relatedness must therefore shape the split before model selection. Sequence similarity clusters should remain atomic, complete species or orders should be reserved for external evaluation when sample size permits, and performance should be reported against distance to the nearest training sequence. Protein benchmarks reach the same conclusion: extrapolation-oriented splits reveal limitations that random partitions conceal [36,61,62].

### 2.3. Genome and Transcript Evidence Are Uneven

Olfactory genes are difficult not only because their proteins diverge but also because their loci are difficult to assemble and predict. Tandem clusters may collapse, heterozygosity can create apparent duplicates, short exons may be missed, pseudogenes may be converted into coding models, and predictors can split one receptor or fuse adjacent copies. Global completeness scores such as BUSCO provide useful context but do not guarantee correct reconstruction of a rapidly evolving local cluster [63]. BRAKER3 can integrate RNA-Seq and protein evidence, yet its output remains conditional on evidence available at each locus [64].

Transcriptomes provide important support but sample biology selectively. Antennal expression varies with sex, stage, circadian state, blood feeding, mating, host exposure, and the sensory appendage examined [65,66,67,68,69,70,71,72,73,74,75,76]. Failure to observe a transcript is not evidence that a gene is absent, and antennal enrichment is not proof of olfactory function. A sequence model may score a plausible locus without transcript support, but it cannot determine whether that locus is expressed, intact, or behaviorally relevant.

The sequence supplied to an SFM is therefore already an interpretation. A PLM inherits coding-model errors, whereas a DNA model may receive a window containing introns, repeats, neighboring genes, or a truncated coding region. Controlled truncations, exon removal, frameshifts, and synthetic fusions can test robustness, but real manually corrected loci are also needed. Every benchmark record should retain assembly and annotation versions, genomic coordinates, transcript support, protein sequence, partial status, and manual corrections so that upstream errors are not mistaken for model behavior.

### 2.4. Conventional Pipelines Define Both the Baseline and the Failure Map

Conventional evidence remains essential for candidate retrieval and verification. Local sequence searches identify interpretable neighbors, profile HMMs summarize family conservation, Pfam and InterPro annotate domains, topology and signal peptide methods constrain architecture, and phylogenetic analysis tests family or orthology assignments [10,11,12,13,14,15,16,17,18,19,20]. Gene structure, synteny, expression, and manual inspection resolve errors that a sequence classifier cannot. Curated resources, such as iORbase and arthropod genome initiatives, add useful references, although taxonomic coverage and validation depth remain uneven [77,78].

**Figure 1:**
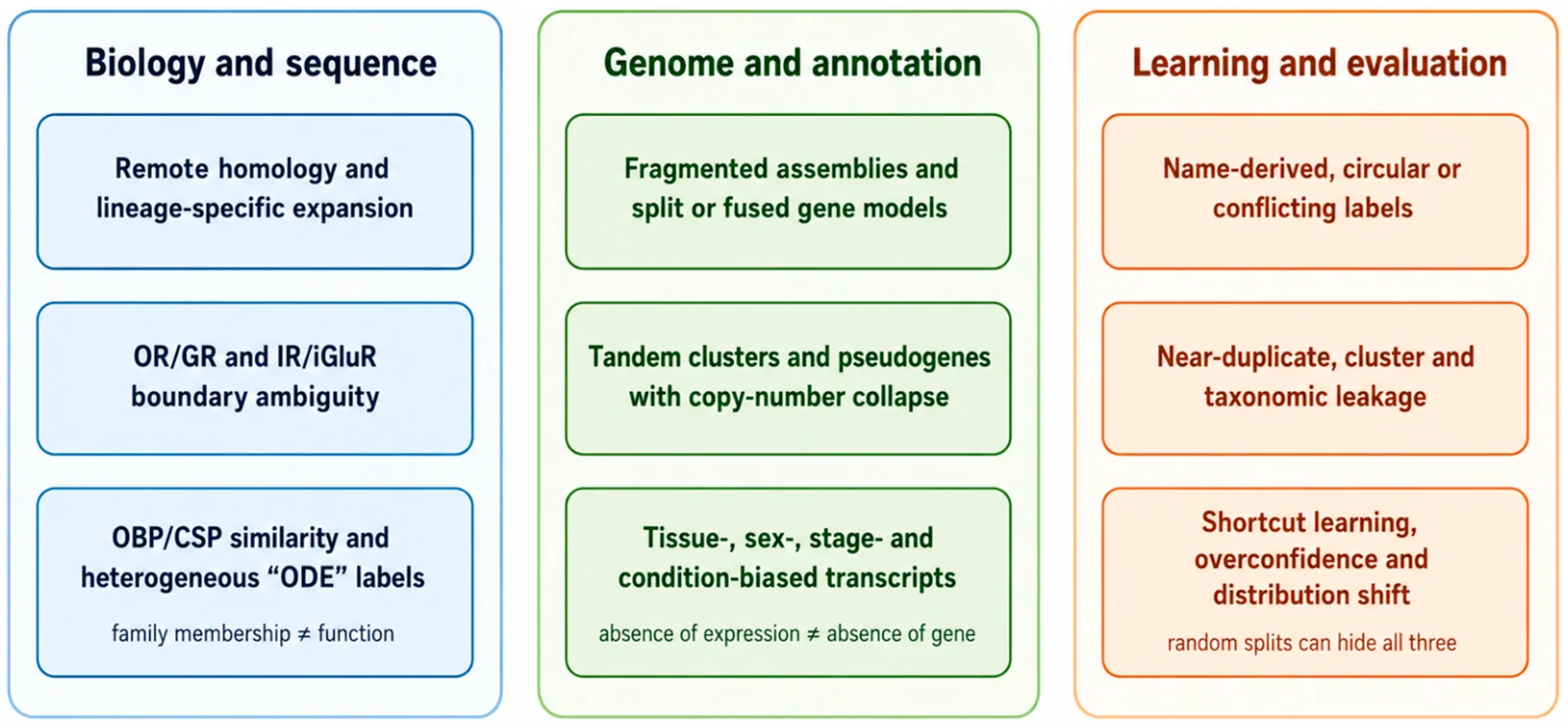
Failure layers in insect olfactory gene mining. Biological divergence, genome annotation errors, and learning or evaluation artifacts can produce similar apparent model errors. A benchmark that samples only one layer cannot establish deployment reliability.

The useful contribution of an SFM appears at a named decision point: retrieving candidates missed by a conservative profile, re-ranking weak hits, comparing alternative gene models, or prioritizing sequences for follow-up. BLAST and HMMER are therefore both baselines and complementary evidence. The comparison should ask whether an SFM-augmented workflow improves the same decision on the same frozen split. Performance in isolation says much less. The main failure layers are summarized in Figure 1.

Table 1 links the major biological and technical failures to diagnostic tests and benchmark safeguards. The table also states the interpretation boundary for each test because detecting a plausible sequence, assigning a family, and establishing sensory function are different outcomes.

## 3. What Sequence Foundation Models Can and Cannot Add

### 3.1. DNA and Protein Representations Address Different Units of Inference

DNA foundation models process nucleotide sequences and may capture motifs, coding grammar, splice signals, codon context, and genomic neighborhoods [25,26,27,28,29,30,31,32,33,79,80,81,82,83]. Transformer encoders, long-convolution systems, and state-space architectures offer different trade-offs between resolution, context length, and computation. Long context may help evaluate gene boundaries or tandem clusters, but the ability to read a long sequence does not guarantee attention to the relevant locus. Training predominantly on vertebrate or microbial genomes may also limit transfer to non-model insects.

PLMs operate on predicted amino acid sequences and are more directly aligned with protein-family retrieval, remote homology, topology, and structural constraint [22,23,24,84]. They remove synonymous nucleotide variation but inherit every coding error. Embeddings can support retrieval and classification, while sequence likelihoods or residue-level scores can flag unusual regions. However, an embedding is a high-dimensional summary, not an explanation. Family separation may reflect length, hydrophobicity, taxonomy, signal peptides, or database conventions rather than a causal biological feature.

The two representations can provide complementary checks. A DNA model evaluates the coding locus and surrounding context, whereas a PLM evaluates the translated product. Agreement with gene structure, domains, topology, and phylogeny strengthens a candidate; disagreement identifies a locus for review. Here, topology means the number and orientation of membrane-spanning segments, whereas structural compatibility means consistency with a predicted three-dimensional fold or conserved family architecture. The terms are related but not interchangeable.

### 3.2. Candidate Ranking and Evidence Integration Are the Credible Near-Term Uses

Candidate triage is the most plausible first use. Representation-space similarity can rank open reading frames or predicted proteins beyond the leading BLAST or HMM hits, leaving a manageable set for inspection. Deep learning-based protein-family classifiers already complement alignment-based annotation in broad settings [84]. Whether this advantage transfers to OR, IR, OBP, CSP, or SNMP annotation remains an empirical question that requires family-specific profiles and hard negatives.

Alternative gene models offer a second use. When two translations differ in length, topology, or conserved features, a PLM can help rank the version that merits closer inspection, while a DNA model contributes splice and coding-context evidence. The result remains a hypothesis. Original coordinates, rejected alternatives, transcript support, and the reason for the choice must remain available to the curator.

Evidence integration is operationally defined here as a pipeline that retains distinct evidence sources while using them to rank or triage candidates. A conventional search may provide BLAST or HMM scores; an SFM may add embedding similarity or a classifier probability; independent features may include domains, topology, gene structure, phylogenetic placement, antennal expression, and assay evidence. Integration can be implemented as a transparent rule-based sequence of filters or as a validation-trained stacking model. In either case, each component remains reportable, thresholds are fixed on validation data, and a family score cannot be promoted to a functional claim. Multiplying correlated scores without calibration would create false precision.

**Figure 2:**
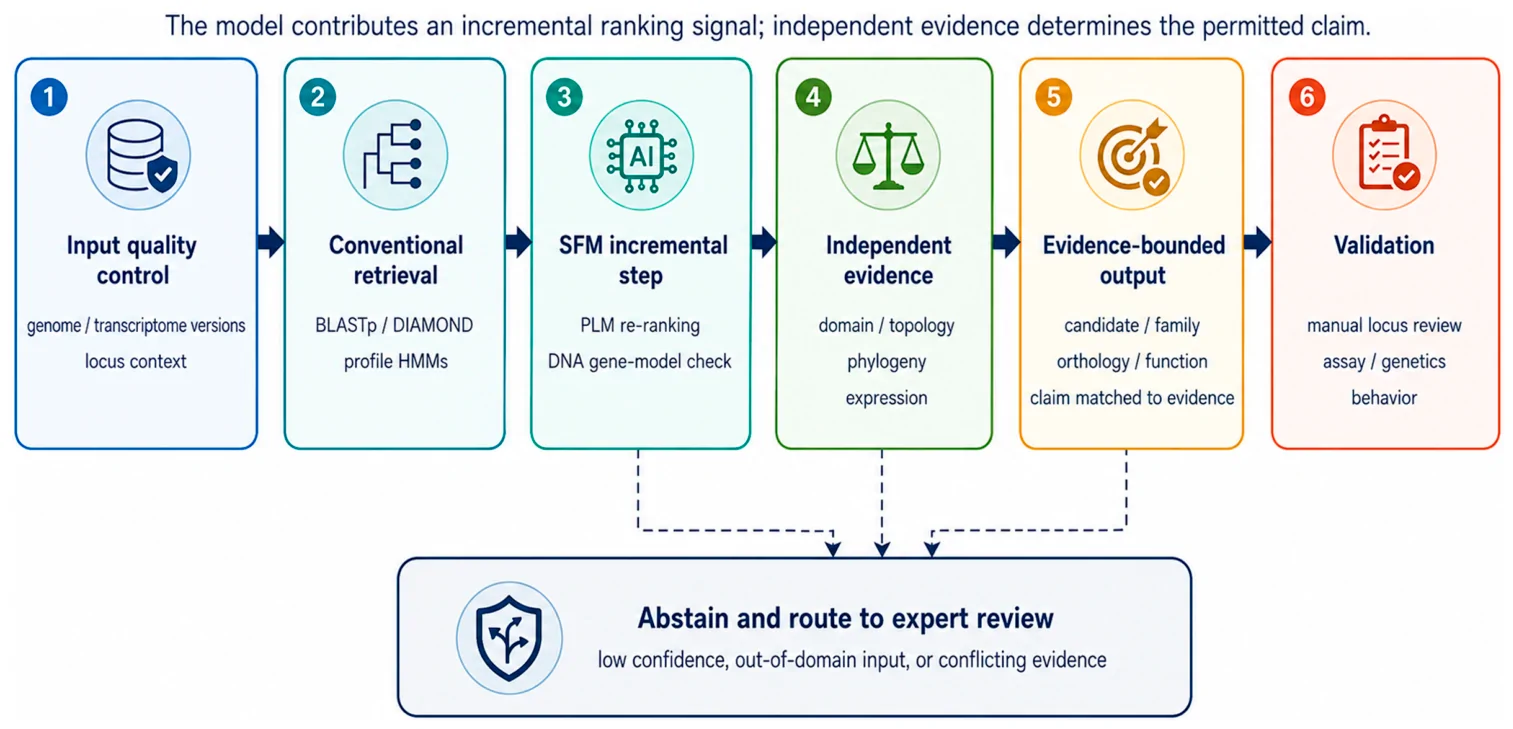
Evidence-constrained use of sequence foundation models in insect olfactory gene discovery. Conventional retrieval establishes the baseline; a PLM or DNA foundation model contributes an incremental ranking or gene-model signal. Independent domain, topology, phylogenetic, expression, and assay evidence determines the permitted claim. Low-confidence, out-of-domain, or conflicting cases are routed to expert review.

Low-label transfer is attractive when few genes have been curated in a target species. Frozen embeddings, linear probes, and parameter-efficient adaptation reduce trainable parameters [22,23,24,36,61,85]. A few-shot experiment is informative only when target examples are not close paralogs of the test sequences and the final holdout remains untouched during threshold selection. The same target examples should also be added to the HMM profile or nearest-neighbor reference set. Otherwise, an apparent gain may come from the extra labels rather than pretraining. The evidence-constrained workflow is summarized in Figure 2.

### 3.3. Limitations Remain Even When Representations Are Useful

SFMs differ in pretraining corpora, sequence units, objectives, context lengths, architectures, and documentation. A model exposed to Drosophila melanogaster or close relatives during pretraining is not evaluated under the same novelty condition as one applied to an unseen order. Incompletely documented corpus overlap should be reported as unknown, not converted into a zero-shot claim. Computational requirements, checkpoint availability, sequence-length limits, and software dependencies also affect whether a method is practical for routine curation.

Interpretability is a second limitation. Attribution maps, attention weights, nearest neighbors, and residue perturbations may identify candidate regions, but they do not by themselves establish mechanism. A plausible interpretation should survive controls for length, composition, topology, and taxonomy and should agree with independent biological evidence. Similarly, a high confidence score is actionable only within a validated operating domain.

Calibration and abstention are therefore part of model evaluation. Calibration asks whether predicted probabilities match observed error frequencies. Abstention allows the system to leave a sequence unresolved when confidence is low, the candidate is outside the evaluated domain, or evidence conflicts. A system that safely routes difficult loci to review may be more useful than one that assigns every sequence a label.

### 3.4. Representative Case Study: Remote Insect Chemoreceptor Homology

Himmel and colleagues provide the closest published example of PLM-supported analysis in this domain [21]. Their study combined sequence searches, predicted structures, Foldseek-based structural comparison, phylogenetic analysis, and ESM-2-derived embedding conservation scores to place insect chemoreceptors within a much wider cryptic seven-transmembrane superfamily [19,21]. The study shows why representation- and structure-based evidence can be valuable when local sequence identity is weak.

The same study also illustrates the boundary of current evidence. ESM-2 was used as one component of a multi-method evolutionary analysis, not as an autonomous whole-genome annotator. The work did not compare ESM-2, BLASTp, and family HMMs on a frozen cluster- and taxon-aware annotation benchmark, nor did it establish sensory function for newly connected proteins. It therefore supports the plausibility of complementary representation evidence, not a claim that a PLM outperforms conventional annotation.

A benchmark derived from this case would start with experimentally or structurally supported difficult positives selected independently of the evaluated models. The search background would include complete proteomes, OR/GR boundary negatives, unrelated multipass membrane proteins, and open-set families. BLASTp or DIAMOND, profile HMMs, Foldseek, and a frozen PLM would receive identical inputs and splits. Recall at a fixed curator budget, nearest-training identity, OR/GR error direction, calibration, and the number of candidates requiring manual review would then test whether the PLM changes the practical conclusion.

## 4. Evidence Standards and Operational Reference Labels

### 4.1. The Biological Claim Determines the Reference Standard

One sequence can support several statements with varying confidence. We distinguish six tasks: candidate locus detection, family membership, orthology or subfamily placement, expression association, ligand response, and organism-level function. “Gold standard” is often used loosely, but most biological labels are conditional on assay and provenance. We therefore use high-confidence reference set for records that satisfy prespecified task-specific evidence and reserve unresolved status for conflicts or insufficient evidence.

Table 2 defines the minimum evidence for inclusion in each high-confidence evaluation set. Evidence supporting a lower level cannot be carried upward unchanged. A complete OR-like coding sequence may satisfy a family-membership task, but ligand response requires a receptor-assay record with defined compound, concentration, and response criterion. Organism-level function requires perturbation or equivalent causal evidence linked to a phenotype. Orthology should not be assigned from the nearest BLAST hit alone when duplications or losses make one-to-one relationships uncertain.

### 4.2. Evidence Should Be Represented as Assertions, Not a Single Inherited Label

A reproducible record stores separate assertions linking a sequence or locus to a claim, evidence type, source, taxon, assay context, curator, date, and confidence. This assertion-based record structure distinguishes protein-family membership from olfactory function and allows one record to be positive for one task but unresolved for another. Database names can remain as source annotations without becoming independent evidence.

Direct experimental evidence is not automatically uniform. Heterologous expression can demonstrate ligand-dependent receptor activation under specified conditions, but assay system, co-receptor, concentration, and threshold influence the result. Expression data require tissue, stage, sex, condition, and quantification metadata. Genetic and behavioral studies require perturbation design, controls, and phenotype definition [39,40,41,42,43,44,45,46,47,86,87]. Negative assays should be retained because they constrain claims and prevent repeated selective reporting.

Conflicting evidence should remain visible. A full-length protein may have strong family support but weak phylogenetic placement; a short transcript may be antennally enriched but lack a complete coding model; an automated name may disagree with curated topology. Such records can enter challenge sets or manual-review analyses without being forced into the high-confidence test set. The permitted model output includes unresolved or abstain.

### 4.3. Label Audits Are Independent of Model Performance

Reference data require checks for exact duplicates, near duplicates, fragments, contamination, sequence-name inconsistency, taxonomic errors, and incompatible evidence. Similarity clustering is performed before splitting. Family-specific reviewers should inspect a sample of positives, boundary negatives, and the highest-risk records without seeing model predictions whenever feasible. Corrections are versioned so that later database updates do not silently change an earlier evaluation.

Name-derived labels are particularly risky. If a record was labeled by BLAST or a profile HMM and then used to claim that a new model outperforms those methods, the comparison is circular. The benchmark should either require independent evidence for the high-confidence test set or describe the outcome more narrowly as agreement with existing annotation. Label provenance is therefore part of the target variable, not administrative metadata.

## 5. A Benchmark Agenda for Insect Olfactory Sequence Models

### 5.1. Existing Frameworks Provide Principles but Not This Domain-Specific Task

Several established frameworks inform the agenda. TAPE tests transfer from protein pretraining to structural and fitness-related tasks [36]. FLIP and ProteinGym emphasize extrapolation across protein fitness landscapes and experimentally measured variants [61,62]. BEND and later DNA model comparisons evaluate genomic representations on gene finding, regulatory annotation, and other predominantly human tasks [34,35]. DOME provides reporting recommendations for supervised machine learning in biology [88]. These resources establish useful practices, including frozen tests, meaningful baselines, and task-specific metrics.

They do not directly represent the combined problem addressed here: retrieving rapidly evolving insect gene families from realistic genomic backgrounds, separating close family boundaries, auditing fragmented gene models, assigning evidence-coded labels, and testing transfer across species or orders. The proposed increment is therefore not a new generic machine learning principle. It is a domain-specific composition of existing principles around olfactory annotation failures and the biological claims that curators and experimentalists actually make.

Recent studies also show why task definition matters. Domain-specific DNA barcode pretraining can outperform general DNA models on insect taxonomic identification, but taxon classification is not gene-family annotation [89]. SegmentNT predicts nucleotide-level genomic elements and demonstrates cross-species gene-annotation transfer, yet it does not resolve insect olfactory-family boundaries [90]. Protein models trained with text annotations can improve broad function retrieval, but text-derived labels introduce a different provenance problem [91]. Table 3 summarizes what these frameworks establish and what remains untested for insect olfactory discovery.

### 5.2. Data, Splits, and Baselines Must Be Frozen Before Model Selection

The benchmark should be organized around annotation decisions rather than model families, meaning broad method classes such as similarity search, profile HMM, PLM, or DNA foundation model. Five tasks cover most of the workflow: candidate retrieval from a realistic background; family-boundary classification against biological alternatives; orthology or subfamily assignment under phylogenetic holdout; detection of problematic gene models; and where evidence permits, receptor-ligand or enzyme-substrate ranking. Combining these tasks into one score would obscure differences in label quality and biological meaning.

Each record should include stable identifiers, sequence hashes, coordinates, taxon, assembly and annotation versions, completeness, evidence assertions, and cluster assignment. Positives are selected independently of the tested methods. Negatives are task-specific: GRs and other multipass proteins for ORs; conserved iGluRs for IRs; secretion- and length-matched proteins for OBPs and CSPs; other CD36 proteins for SNMPs; and metabolic paralogs for ODE-related tasks. Unrelated proteins and held-out families test open-set rejection but do not replace boundary negatives.

Similarity clusters are allocated atomically to train, validation, and test partitions. Validation data are used for hyperparameters, thresholds, calibration, and model selection. The test set remains frozen and is evaluated once for the primary report. Additional species-, order-, and time-based holdouts answer different questions and should not be collapsed. Exact hash overlap, nearest-neighbor identity, taxonomic overlap, and known pretraining exposure are reported for every split [92,93,94,95].

**Figure 3:**
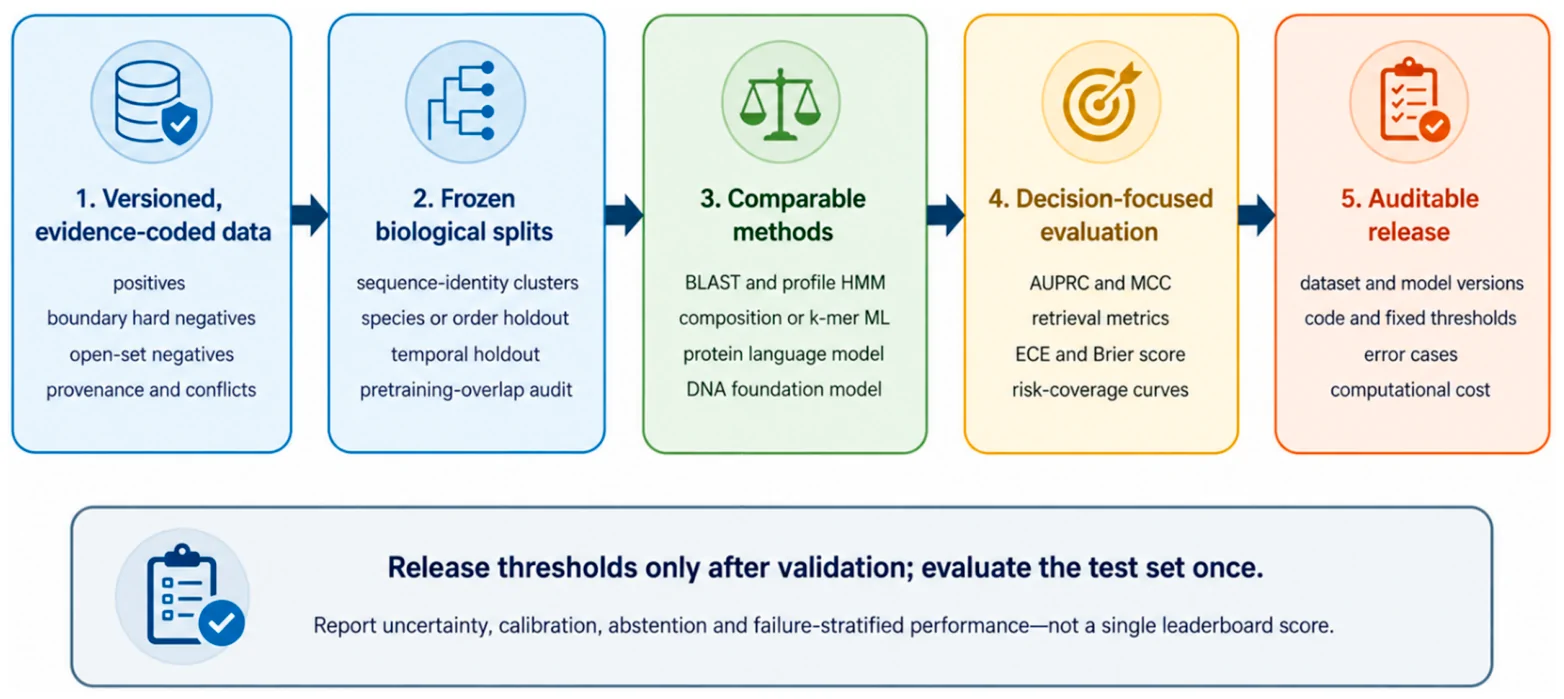
Minimum benchmark architecture for insect olfactory sequence models. Versioned evidence-coded data, frozen biological splits, comparable conventional and learned methods, decision-focused metrics, and an auditable release form the core design. Thresholds are selected on validation data before a single final test evaluation.

All methods receive the same records and split. Minimum conventional baselines include BLASTp or DIAMOND, family-specific profile HMMs, domain and topology rules, and simple length, composition, or k-mer models [10,11,12,13,14,15,16,17,18,19,20]. SFM conditions specify checkpoint, layer, pooling, frozen or fine-tuned status, trainable parameters, and random seeds. Hybrid systems may combine conventional and SFM features, but their incremental value is measured against the best component under the same validation policy. The minimum benchmark architecture is summarized in Figure 3.

### 5.3. Metrics Should Reflect Imbalance, Ranking, Uncertainty, and Workload

Olfactory datasets are imbalanced, and errors have unequal biological costs. Macro-averaged F1 and the Matthews correlation coefficient prevent large families from dominating summaries. Area under the precision-recall curve is generally more informative than AUROC for rare positives. Per-family sensitivity, specificity, and boundary-specific confusion matrices should be accompanied by cluster- or taxon-level confidence intervals.

Retrieval metrics include recall at a fixed k, mean reciprocal rank, normalized discounted cumulative gain when relevance is graded, and recall at a fixed inspection budget. Probabilistic evaluation includes Brier score, negative log-likelihood, reliability diagrams, and expected calibration error [95,96]. Risk–coverage curves quantify selective prediction: they show how error changes as the system abstains on a larger fraction of candidates [97].

Uncertainty resampling should follow the dependence structure. Sequences within one evolutionary cluster are not independent, so bootstrap sampling operates on clusters or taxa rather than individual sequences. Repeated random seeds describe optimization variability but do not provide biological replication. Pairwise method comparisons should use the same held-out clusters and report confidence intervals for differences. A ranking that changes by taxon, identity threshold, or split does not support a universal superiority claim [93,94].

### 5.4. A Practical Workflow Makes the Agenda Executable

Table 4 translates the agenda into a reproducible pipeline. Tool names are examples rather than mandated choices; versions, parameters, databases, and licenses must be reported. The central design rule is that the SFM enters as a named comparator or ranking component, while independent biological evidence remains available for verification.

This workflow also clarifies training inputs and objectives. A PLM retrieval condition may be frozen and use nearest-neighbor distance in embedding space, or it may train a linear classifier on family labels. A DNA model may be fine-tuned to classify genomic windows or segment gene elements. A hybrid ranker may use validation data to combine HMM, PLM, topology, and expression features. These are different experiments and should be reported separately rather than described collectively as “using an SFM”.

### 5.5. Error Analysis, Prospective Validation, and Governance Complete the Benchmark

Error analysis follows the failure map: family, nearest-training identity, taxonomic distance, sequence completeness, cluster size, evidence level, and open-set status. Review of false positives, false negatives, and high-confidence errors may reveal incorrect database labels or gene models. Such cases should be separated from genuine prediction errors and retained in a versioned correction log.

Prospective validation provides the clearest utility test. A target insect and ranking criteria are chosen before model application. Conventional and SFM-augmented workflows produce fixed review lists. Additional curated genes recovered, validated hits per assay, review time, and recall at a fixed budget then become primary outcomes. Negative assays and unresolved loci remain part of the record. If the SFM-augmented workflow does not improve yield or workload, added complexity has not produced practical value.

A staged release keeps claims aligned with evidence. Release 1 can address protein-family retrieval and boundary classification using curated proteins, explicit hard negatives, cluster-aware splits, and BLAST, HMM, simple-feature, and PLM baselines. Release 2 can add genomic loci, alternative gene models, transcript support, partial genes, and controlled corruption tests for DNA models. Release 3 should add receptor-ligand, enzyme-substrate, genetic, and behavioral tasks only where assay-specific labels are sufficient. Sparse functional records should not be diluted by family labels.

Governance makes these releases auditable. Every version should identify source databases, extraction dates, evidence rules, sequence hashes, taxonomic mappings, cluster parameters, model versions, and changes from previous releases. A public development set can be paired with a hidden or refreshed post-cutoff test to limit repeated optimization and pretraining exposure. Biological curators and machine learning researchers should jointly review label rules, leakage audits, and error cases. A smaller defensible benchmark is preferable to a larger dataset whose labels and splits cannot support the stated claim.

## 6. Cross-Species Transfer and Pest Management Relevance

The route from bioinformatics to pest management is necessarily staged. An SFM may first contribute to an auditable candidate repertoire for a species of agricultural or medical importance. Domains, topology, phylogeny, locus structure, and expression then reduce that repertoire to receptor-ligand or enzyme-substrate hypotheses. Lure design, repellents, mating disruption, and related interventions enter only after functional and behavioral validation.

Cross-species transfer is most attractive for newly assembled pests, invasive species, vectors, parasitoids, and beneficial insects with sparse references. Zero-shot embeddings may provide a common retrieval space, while a small set of curated target genes can support parameter-efficient adaptation [85]. The target taxon remains a holdout until the adaptation protocol is fixed, and zero-shot and few-shot results are compared with conventional methods given the same target examples.

Transfer also creates risk. A model optimized on Drosophila and mosquitoes may fail in distant orders or in species with unusual repertoire evolution. Functional overprediction is particularly consequential for pollinators and natural enemies, where a proposed intervention target may be non-specific or shared with beneficial insects. Cross-taxon selectivity, concentration, toxicity, and behavior remain unresolved by a sequence score.

The practical objective is a smaller, better-ordered workload. In a new beetle or moth genome, a hybrid workflow might recover OR or IR candidates missed by conservative profiles, flag suspicious models in tandem clusters, and rank genes with antennal expression. The relevant outcomes are additional curated genes, assays avoided, review time saved, or validated hits gained at a fixed budget. These measures connect molecular bioinformatics to application without treating a computational prediction as a pest control technology.

## 7. Conclusions

Sequence foundation models may improve remote candidate retrieval, review of ambiguous gene models, and transfer to poorly annotated insects. Their value is not established by model scale, visually separated embeddings, or random-split accuracy. It requires an incremental gain over BLAST or DIAMOND, profile HMMs, domains, topology, phylogeny, and simple baselines under cluster-aware and taxonomically realistic evaluation, with calibrated uncertainty and task-specific high-confidence reference labels.

In the near term, SFMs are best treated as components for retrieval, ranking, and evidence organization. A useful system should produce a well-ordered candidate list, preserve the independent evidence behind each decision, and identify cases that remain unresolved. The proposed agenda makes that role testable while maintaining a clear boundary between candidate detection, family assignment, functional inference, and experimental validation.

## Figures and Tables

**Table 1:** Failure modes, diagnostic tests, and benchmark safeguards for insect olfactory gene discovery.

Failure Mode	Diagnosis	Required Safeguard	Interpretation Boundary
Divergent tuning ORs	Weak local similarity; inspect nearest identity, HMM score, topology, structure, and phylogeny.	Stratify by distance to training data; include difficult positives supported independently.	A high SFM score supports retrieval, not ligand function.
OR/GR boundary	Both are divergent multipass chemoreceptors with partly shared architecture.	Use taxon-matched ORs and GRs as reciprocal hard negatives; report both error directions.	Family separation requires phylogenetic and structural support.
IR/iGluR boundary	Conserved iGluR domains can dominate the signal.	Evaluate co-receptors, tuning IRs, and conserved iGluRs separately under cluster-aware splits.	An iGluR-like score does not establish a chemosensory IR.
OBP/CSP and atypical soluble proteins	Length, signal peptides, and cysteine patterns are informative but imperfect.	Match negatives by length and secretion status; include partial and atypical proteins.	Sequence class does not establish olfactory expression or transport.
Heterogeneous ODE labels	ODE combines unrelated enzyme families and often inherits expression- or name-based labels.	Separate enzyme family from odorant-degradation evidence; include metabolic paralogs.	Enzyme-family membership is not evidence of odorant degradation.
Fragmented or fused gene models	Assembly breaks, tandem arrays, pseudogenes, and prediction errors alter the input.	Use real corrected loci plus controlled truncation, frameshift, split, and fusion stress sets.	A model can flag plausibility but cannot repair a locus without genomic evidence.
Circular or name-derived labels	Database names may copy earlier homology predictions.	Record evidence codes and maintain an independently supported high-confidence test set.	Agreement with a circular label does not validate biology.
Cluster or taxonomic leakage	Recent paralogs, shared taxa, or annotation pipelines cross partitions.	Allocate clusters atomically; add species/order and temporal holdouts; audit hashes and identity.	Random-split performance cannot support cross-species claims.
Shortcut learning and overconfidence	Models exploit length, composition, taxonomy, or pipeline artifacts.	Use matched negatives, counterfactual controls, calibration, and risk–coverage analysis.	Confidence is actionable only within a validated domain.

**Table 2:** Task-specific criteria for high-confidence reference sets and permitted inference.

Task	Minimum Positive Evidence	Task-Specific Negatives	Conflict or Uncertainty Rule	Permitted Inference
Candidate locus	Genomic coordinates plus coherent coding evidence from transcript, protein homology, or manual reconstruction.	Decoy loci, intergenic sequence, and known noncoding or artifactual models.	Partial or assembly-limited loci are flagged; absent assembly sequence is not a model error.	A locus merits further annotation.
Family membership	Full or explicitly partial sequence with convergent domain/topology, profile, structural, and phylogenetic support.	Closest biological alternatives such as GR for OR or conserved iGluR for IR.	Conflicting family evidence becomes unresolved or a challenge case.	The sequence belongs to a defined family.
Orthology or subfamily	Phylogenetic placement with suitable sampling, duplication/loss assessment, and synteny where informative.	Paralogs from the same and related taxa.	Unstable trees or insufficient sampling permit only broader clade assignment.	Orthology or subfamily placement, not function.
Expression association	Replicated, quality-controlled tissue or cell expression with sex, stage, and condition metadata.	Relevant non-sensory tissues and negative observations with adequate power.	Non-detection is recorded as missing or context-specific, not gene absence.	Association with a sampled sensory context.
Ligand response	Defined receptor/ligand assay with concentration, co-factors, response threshold, controls, and reproducibility.	Tested inactive compounds and assay controls.	Assay-system disagreement remains assay-specific; no forced consensus label.	Response under the stated assay conditions.
Organism-level function	Genetic or equivalent causal perturbation linked to a controlled physiological or behavioral phenotype.	Sham, genotype, vehicle, and relevant alternative-pathway controls.	Pleiotropy or inconsistent phenotypes limit the claim and trigger review.	A causal role in the measured phenotype.

**Table 3:** Relationship between existing sequence-model benchmarks or studies and the proposed insect olfactory agenda.

Framework or Study	Data and Objective	Labels or Outputs	Relevant Contribution	Remaining Gap for Insect Olfaction
TAPE [36]	Protein pretraining evaluated by downstream transfer.	Structure and fitness-related tasks.	Comparable transfer tasks and standard baselines.	No genomic retrieval, insect family boundaries, or evidence hierarchy.
FLIP/ProteinGym [61,62]	Protein fitness landscapes and experimentally measured variants.	Continuous fitness or variant effects.	Extrapolation-oriented splits and assay-grounded targets.	Mutation fitness is not gene-family retrieval or locus reconstruction.
BEND and DNA benchmarks [34,35]	DNA representations on gene finding, regulatory, expression, and variant tasks.	Genomic elements and task-specific outcomes.	Biologically meaningful tasks and matched representation comparisons.	Predominantly human tasks; no PLM comparison or olfactory hard negatives.
BarcodeBERT/SegmentNT [89,90]	Invertebrate barcode pretraining or nucleotide-level genome segmentation.	Taxonomy or genomic element labels.	Domain-specific pretraining and cross-species evaluation.	Taxon identity and generic gene elements do not resolve olfactory families.
Himmel et al./PAIR [21,91]	Multi-method chemoreceptor homology or text-augmented protein representation.	Evolutionary relationships or broad function labels.	Direct PLM-supported insect example and evidence that annotation text changes representations.	No frozen insect olfactory BLAST/HMM/PLM annotation comparison.
Proposed agenda	Versioned loci/proteins, hard negatives, cluster and taxon holdouts, and evidence assertions.	Retrieval, family, orthology, gene-model, and assay-specific tasks.	Connects failure modes, claim levels, abstention, and curator workload.	Prospective specification; requires independent implementation and validation.

**Table 4:** Practical workflow for an evidence-constrained insect olfactory benchmark.

Step	Decision	Example Tools or Methods	Required Output	Interpretation Boundary
1	Input quality	Assembly statistics, BUSCO, BRAKER3, transcript/protein support [63,64].	Versioned assembly, GFF, CDS/protein, coordinates, partial status.	Global completeness does not prove local cluster accuracy.
2	Conventional retrieval	BLASTp, DIAMOND, MMseqs2, family HMMs [10,11,12,15,20].	Ranked hits with scores, coverage, identity, and nearest reference.	Similarity supports candidacy; thresholds define baseline recall.
3	Architecture checks	Pfam/InterPro, SignalP 6.0, topology prediction [13,14,18].	Domains, secretion status, transmembrane topology, exceptions.	Architecture constrains family assignment but does not prove function.
4	SFM comparator	Frozen ESM/ProtT5 retrieval, linear probe, or DNA model locus scoring [22,23,24,25,26,27,28,29,30,31].	Checkpoint, pooling, training labels, objective, probability or rank.	Each SFM condition is a separate experiment.
5	Evolutionary and locus verification	MAFFT, IQ-TREE 2, Foldseek, synteny, genome browser [16,17,19].	Tree placement, structural neighbor, cluster context, alternative model review.	Unstable placement remains unresolved.
6	Expression and functional evidence	RNA-Seq or targeted expression; heterologous, genetic, and behavioral assays.	Evidence assertion with tissue, condition, assay, controls, and negatives.	Expression is association; assay evidence is context-specific.
7	Integration and reporting	Rule-based triage or validation-trained stacking; calibration and abstention.	Ranked candidates, component evidence, uncertainty, review route, workload.	No component may support a stronger claim than its evidence level.

## Data Availability

No new empirical data were created or analyzed in this review. The literature, databases, software, and benchmark resources discussed in this article are publicly available from the cited sources where applicable. The figures and benchmark recommendations are conceptual and do not represent a completed empirical comparison.
